# Special Issue “From Basic Science to Treatment Strategies: Personalized Cancer Therapy”

**DOI:** 10.3390/ijms25031435

**Published:** 2024-01-24

**Authors:** Meri Muminovic, Atif Hussein, Luis E. Raez

**Affiliations:** 1 Memorial Healthcare System, Division of Hematology and Oncology, Pembroke Pines, FL 33028, USA; 2 Memorial Cancer Institute, Division of Hematology and Oncology, Hollywood, FL 33021, USA; ahussein@mhs.net; 3 Memorial Cancer Institute, Division of Hematology and Oncology, Pembroke Pines, FL 33028, USA; lraez@mhs.net

Molecular testing has created a revolution in cancer. Thanks to several new technologies, we have been not only able to find genes and biomarkers with predictive and prognostic value, but we have also been able to better classify tumors and find efficacious targeted therapies. In this current series, we conduct a comprehensive review of several genetic aberrations and the role that they play in finding specific therapies for several types of malignancies.

Cancer is a neoplastic process that is driven by somatic and germline or heritable mutations. Genetic testing can be completed on the blood, saliva or even the tumor itself. Gene expression profiling (GEP) is the most ample and essential resource for the management of cancers. GEP allows us to measure the level of gene expression and to gain more insight into tumor characteristics, prognosis and risk of recurrence [1]. The sequencing allows for the discovery of driver mutations that lead the oncological pathogenesis and other byproduct mutations that do not directly affect the growth of cancer [2].

Until recently, germline testing was the standard of care mainly for both breast and ovarian cancer. Germline genetic testing is used to discover heritable mutations that can increase the familial risk of cancer and help make future treatment decisions. Human epidermal growth factor receptor-2 (HER-2) and BRCA 1/2 were tested previously and have become targets for future oncological therapeutics, such as poly ADP ribose polymerase (PARP) inhibitors, that are able to treat a wide variety of different oncological conditions such as breast, ovarian, peritoneal, pancreatic and prostate cancers. Germline testing has ultimately paved the wave to start somatic testing.

Somatic mutations, unlike germinal mutations, are thought of as random and non-inherited. If the mutation occurs in the somatic tissue, unless there is another concurrent mutational occurrence, the mutation may remain silent. Somatic testing has become the standard of care in the diagnosis and treatment of many solid oncological malignancies, including lung cancer. Somatic mutational profiling has transformed the diagnosis and management of a once lethal disease, non-small cell lung cancer (NSCLC), to a manageable condition. The driver mutations in NSCLC such as EGFR, KRAS, ROS1, ALK and HER2 are the most common mutations that have been discovered via somatic mutational profiling and have made targeted therapy an option for many patients (see the clinical and molecular reviews in this Special Issue of the journal).

In this Special Issue of the *International Journal of Molecular Sciences*, we include numerous reviews on molecular testing and targeting of mutations that are vital to cancer diagnosis and treatment. We evaluate and report on different genes and pathways, including NTRK, HER-2, BRAF/MEK, EGFR, ALK, ROS1, IDH, FGFR, BTK, PI3K, folate receptor alpha, hypoxia inducible factor and CDK4/6, that are important therapeutic targets in the combat of cancer. Lastly, we also report on new innovative diagnostic techniques from solid to liquid biopsies, including circulating tumor DNA.

Molecular testing is a constantly changing field that allows for the detection of driver mutations. The different testing modalities include DNA sequencing, IHC, FISH, Sanger, NGS, and whole-exosome and whole-genome sequencing. DNA sequencing is the oldest testing modality with the lowest sensitivity due to the need for high tumor cellularity for adequate testing. Immunohistochemistry (IHC) is both highly sensitive and specific and allows rapid results to be obtained. FISH is a test that evaluates the location and total number of chromosomes that are in both dividing and non-dividing cells. It uses a variety of colors that bypass each other as genes are being pulled apart. It allows for the discovery of translocations, duplications, deletions and microdeletions. The Sanger method uses modified nucleotides to cease the replication process when it encounters a false nucleotide and allows for nucleic acids of various lengths to accumulate; this method is able to evaluate multiple genes or whole genomes [3]. NGS hybrid (DNA/RNA) testing allows us to evaluate multiple genes and even genomes. NGS testing has high sensitivity and allows for testing in different sites such as the blood, i.e., circulating tumor DNA (ctDNA). Lastly, whole-exosome sequencing (WES) and whole-genome sequencing (WGS) have transformed molecular testing in cancer. WES is able to evaluate genome sequences that code for proteins which allow for the detection of mutations, without evaluating the whole genome. Meanwhile, WGS is able to solve different variants of protein-coding regions with expansive coverage of exons and variants that may alter gene expression, splicing and chromosome replication [4].

Throughout the years, there has been difficulty in diagnosing cancer patients due to a lack of tissue or adequate samples available at the time. As the evolution of genetic testing has advanced, we have now been able to expand testing to include screening for circulating tumor DNA (ctDNA) and cfRNA. Liquid biopsies allow for low-cost and non-invasive testing, even when there is minimal tumor present. It allows for the monitoring and detection of disease relapse. Although, there are some limitations such as high false negative rates and presence of variable amounts of ctDNA present in the blood, it allows for more adequate testing and can complement NGS testing. There are many different technologies available to examine DNA or RNA, depending on where we find the source of nucleotides like exosomes, circulating tumor cells or platelets, all of which are making important contributions not only to the field of cancer but also to other areas of medicine.

In conclusion, molecular testing has revolutionized the practice of clinical oncology. Initially, testing began with germline testing and advanced to somatic testing involving different testing modalities. Each testing modality is unique, with both positive and negative aspects, and can complement one another to achieve an adequate diagnosis and ultimately the best management for the patient. As more comprehensive molecular testing is being developed, it is becoming more useful in allowing us to discover new targeted therapies for cancer therapy.
